# Incidence and burden of comorbid pain and depression in patients with chronic rhinosinusitis awaiting endoscopic sinus surgery in Canada

**DOI:** 10.1186/s40463-017-0205-3

**Published:** 2017-03-27

**Authors:** Bassem M. N. Hanna, R. Trafford Crump, Guiping Liu, Jason M. Sutherland, Arif S. Janjua

**Affiliations:** 1 Otolaryngology – Head and Neck Surgery, University of British Columbia, Vancouver, BC Canada; 2 Department of Surgery, University of Calgary, Calgary, Canada; 3 Centre for Health Services and Policy Research, School of Population and Public Health, University of British Columbia, 201 – 2206 East Mall, V6T 1Z3 Vancouver, Canada

**Keywords:** Chronis sinusitis, Depression, Endoscopic sinus surgery, Pain, Patient-reported outcomes, Sleep, Quality of life

## Abstract

**Background:**

This study sheds important light on the association between sino-nasal symptoms and global quality of life in patients with chronic rhinosinusitis waiting for endoscopic sinus surgery. Using patient-reported information collected pre-operatively, the primary objective was to report on patients’ pre-surgical sino-nasal symptoms and their association with self-reported pain and depression. The secondary objective was to report on levels of depression and pain among patients in the sample reporting severe sleep problems.

**Methods:**

This is a cross-sectional study of patient-reported outcomes collected prospectively from a cohort of 261 patients assigned to the wait list for elective endoscopic sinus surgery in a large urban region of Canada.

**Results:**

Younger patients and patients with other medical comorbidities were most likely to report significant symptoms of chronic rhinosinusitis and substantial associated pain and depression. In the primary analyses, patients reporting significant symptoms of chronic rhinosinusitis were more likely to report moderate depression or high pain (*p* < 0.01). Subsequently, chronic rhinosinusitis patients with severe sleep problems were 82% likely to report moderate or severe depression and pain.

**Conclusion:**

Preoperative management of depression and pain may be considered in order to improve the health-related quality of life of patients waiting for ESS. As depression and pain were highly prevalent, patients with severe sleep problems may be candidates for prioritized access.

**Electronic supplementary material:**

The online version of this article (doi:10.1186/s40463-017-0205-3) contains supplementary material, which is available to authorized users.

## Background

The syndrome of chronic rhinosinusitis (CRS) is a heterogeneous cluster of sino-nasal symptoms associated with mucosal inflammation. First line treatment for CRS includes medical/pharmacological interventions, such as steroids (topical and/or systemic) and antibiotics. It is estimated that more than 5% of the Canadian population suffer from CRS-related symptoms, resulting in almost one million prescriptions per year [1, 2] and cause significant demands on provincial health care systems.

Many patients with CRS eventually fail pharmacological intervention and progress to endoscopic sinus surgery (ESS) for treatment. This is problematic for many provincial health care systems, where demand for elective (scheduled) surgery outstrips supply which, in turn, results in substantial wait times for CRS patients. For example, in 2016, Nova Scotia reported the median wait time for ESS of approximately 4 months [3] – similar median wait times have been reported in British Columbia (BC) [4]. Wait times are not unique to ESS, as Canada lags many of its peer countries in access to specialized care [5] – despite significant investments, wait times for elective surgery continue to be a major policy issue for provincial governments [6–8].

To manage those patients awaiting elective surgery, regional health authorities in Canada use wait lists. These wait lists are registries for elective surgeries, recording demographics, diagnosis, surgery and date information – including the date that patients were assigned to the wait list and the date of their scheduled surgery. The Achilles heel of the wait list is that very little is known regarding symptom severity or the health status of those patients in the registry. As previous study into the wait list registry has observed, there is no relationship between the self-reported severity of patients’ symptoms and their level of triage on the wait list [9].

The discordance between the severity of patients’ symptoms and the length of time they wait for surgery is concerning, particularly in the case of CRS. Sino-nasal symptoms have been documented to be associated with significant anxiety and depression [10, 11]; especially in patients without polyps [11], and more than half of patients with CRS reportedly suffer from pain [12, 13]. Dysfunction of sleep and pain have been seen to be inter-related in the presence of depression [13]. However, very little is known about the health status of those patients with CRS on the wait list for ESS, representing a significant knowledge gap that can impair clinical decision making.

To address this knowledge gap, the purpose of this descriptive study is to measure the symptom burden and the prevalence of pain or depression among CRS patients waiting for ESS after failed medical management. Previous work has established a need for this information in order to identify opportunities to improve the health status of waiting patients [14].

To do so, this study uses validated patient-reported outcomes (PROs) designed to systematically measure the severity of symptom and functional impairments. The primary analyses is to measure the association between depression or pain with patients’ self-reported symptoms of chronic rhinosinusitis. The secondary objective is explore emerging findings regarding relationship between self-reported sleep problems with depression or pain [15]. The results from cross-sectional analyses of PROs could be used to identify potential gaps in patients’ care while waiting for ESS and potentially provide insight into policies for refining surgical wait times.

## Methods

PROs were prospectively collected from a cohort of CRS patients assigned to the surgical wait list for ESS in the Vancouver Coastal Health (VCH) Authority, a region encompassing Vancouver, Canada – home to over one million residents. Vancouver Coastal Health is responsible for managing the wait list for elective surgeries performed in its acute hospitals.

The population of potential study participants included all patients newly enrolled on VCH’s wait list for bilateral endoscopic sino-nasal surgery with a surgeon identified diagnosis of ‘chronic sinusitis/nasal polyposis’. Specific surgical codes are provided in Additional file 1. Potential participants were identified from VCH’s wait list registry, which includes patients’ contact information. A VCH surveyor contacted potential participants using a standardized telephone script within 2 weeks of being enrolled on the wait list. To be eligible, patients had to be community-dwelling, 19 years or older, scheduled for surgery at least 14 days from being enrolled on the wait list, and able to respond (with or without assistance) to survey questions in English. Patients agreeing to participate were sent the initial survey package, which included survey instructions, the PRO instruments, and a stamped return envelope.

Comorbidity information was collected from participants using a checklist of common chronic and acute conditions. Age and sex was collected via the wait list from participants and non-participants; these groups were compared based on available demographic information. This study reports on participants’ cross-sectional PROs data collected between September 2012 and April 2016. The University of British Columbia’s Behavioral Research Ethics Board (BREB) approved the study.

### Instruments

A number of different PRO instruments were completed by participants, including the Sino-Nasal Outcomes Test (SNOT)-22, the Patient Health Questionnaire (PHQ)-9, and the PEG, representing pain intensity (P) and interference with enjoyment of life (E) and general activity (G). The SNOT-22 was used to measure the severity of CRS-related symptoms. The SNOT-22 is a widely used instrument that has previously demonstrated strong validity, reliability, responsiveness, and ease of interpretation [16]. As its name implies, it is comprised of 22 items scored from 0 to 5. The scores for each item are aggregated to arrive at a global score that ranges from 0 (i.e., perfect health) to 110 (i.e., worst health). The average SNOT-22 score in a healthy non-symptomatic adult is 7 [17].

Depression in patients was measured using the PHQ-9 [18]. This PRO instrument assesses depression in two dimensions: symptoms and functional impairment. It includes nine items, each of which is scored using a four-point Likert scale ranging from 0 (i.e., “Not at all bothered”) to 3 (i.e., “Bothered nearly every day”). The score for each item is aggregated to arrive at a global score that ranges from 0 to 27. PHQ-9 scores of 10, 15 and 20 represent, respectively, moderate, moderately severe, and severe depression [18].

The PEG was used to measure comorbid pain [19]. The PEG has three items; one representing pain intensity and two items for interference. Each item is scored on a 0–10 scale. The overall score is reported as the average of the three items, and scores greater than three have been indicative of high level of pain [20].

### Statistical analysis

Anonymized data was analyzed using SAS 9.4 (Cary, NC). Age and gender sub-groups were compared between participants and non-participants to ascertain possible participant bias. Univariate analyses of the SNOT-22 score were presented overall, by age group, gender and count of reported comorbidities. Few participants reported more than three chronic health conditions, so comorbidity data was categorized into counts of 0, 1, 2, 3 and greater than 3.

Depression scores (PHQ-9) and pain scores (PEG) were summarized, presented using cut-points validated in the literature [18, 20]. Participants’ depression and pain scores were examined relative to their SNOT-22 scores to provide in-depth insight into associations between CRS symptoms and general health.

Independent multivariate models were used to measure associations between patients’ SNOT-22 score with their PHQ-9 and PEG scores and other variables in the three models, including age, sex and category of count of comorbidity. Residuals were assessed visually to detect departures from the models’ assumptions. *P*-values for the regression models were reported. Since the analyses were exploratory, no attempt was made to adjust *p*-values for multiple comparisons.

A linear model was used to analyze SNOT-22 scores to measure whether there was an association between SNOT-22 scores with PHQ-9 depression or PEG pain scores, after adjusting for age, sex and comorbidities. The PHQ-9 and PEG scores were included in the analysis of covariance model as continuous variables. *P*-values were reported after assessing goodness of fit.

Patients’ sleep quality was evaluated. The SNOT-22 sleep score was calculated as the sum of the items associated with sleep quality, items 11–18. The sleep score ranged from 0 to 40, where a score of 40 represented the worst state of sleep health-related quality of life. Patients’ sleep scores were stratified by cut-points of the PHQ-9 and PEG representing moderate depression and high pain, respectively. For each quintile of SNOT-22 sleep score, mean (and standard deviation) PHQ-9 and PEG scores were calculated.

## Results

The study sample included 261 participants, evenly split between males and females, with the modal age group between 51 and 70 years of age (Additional file 1). There were no significant differences between study participants and patients that did not participate on the characteristics of age or gender which would have indicated selection bias; see Additional file 1 for a summary of participants and non-participants.

Analysis of participants’ SNOT-22 scores are provided in Table 1. The average SNOT-22 score was 42.1. The results from the univariate analyses show that younger age tended to be associated with higher SNOT-22 scores relative to older participants; participants between 31 and 50 years of age had SNOT-22 scores 18 points higher than participants aged 70 years and older (*p* < 0.01). Participants with more comorbidities reported higher SNOT-22 scores than those without – participants with three or more comorbidities reported SNOT-22 scores 28 points higher than those participants without comorbidities (*p* < 0.01). There were no differences in SNOT-22 scores between genders (*p* = 0.16).

**Table 1:** Multivariate analysis of patients’ SNOT-22 scores, adjusting for demographics and comorbidities

Model parameter	Coefficient	Standard error	F-Statistic *P*-Value
Intercept	19.39	4.88	<0.01
Gender			
Male	Reference		
Female	3.50	2.52	0.16
Age group			
< = 30	14.00	6.26	0.02
31–50	18.72	4.67	<0.01
51–70	12.70	4.30	<0.01
70+	Reference		
Number of comorbidities			
0	Reference		
1	4.04	3.41	0.23
2	10.50	3.86	<0.01
3	4.31	4.77	0.36
3+	28.45	4.12	<0.01

Overall, 19.2% of participants reported symptoms associated with clinical depression. As shown in Table 2, depression was unevenly reported among the study’s participants. Those between 31 and 50 years of age reported significantly higher PHQ-9 scores than participants older than 70 years (*p* < 0.01). Participants with more than one comorbidity reported higher levels of depression. Further, participants with three or more comorbidities reported PHQ-9 scores 2.7 points higher than those without any significant comorbidities (*p* < 0.01).

**Table 2:** Multivariate analysis of patients’ PHQ-9 and PEG scores, adjusting for demographics and comorbidities

	PHQ-9			PEG		
Parameter	Coefficient	Std Err.	*P*-Value	Coefficient	Std Err.	*P*-Value
Intercept	2.13	0.21	<0.001	1.35	0.18	<0.01
Gender						
Male	Reference			Reference		
Female	0.97	0.11	0.75	1.13	0.09	0.20
Age group						
< =30	1.52	0.26	0.11	1.61	0.23	0.04
31–50	1.76	0.2	<0.01	1.62	0.17	<0.01
51–70	1.34	0.18	0.10	1.40	0.16	0.03
70+	Reference			Reference		
Number of comorbidities						
0	Reference			Reference		
1	1.03	0.14	0.84	1.33	0.13	0.02
2	1.74	0.16	<0.01	1.72	0.14	<0.01
3	1.61	0.2	0.02	1.83	0.18	<0.01
> 3	3.16	6.58	<0.01	2.39	0.15	<0.01

Among participants, 44.6% reported PEG scores of three or greater, indicating high pain. Pain was highest among participants 50 years of age or less; relative to the oldest age group, this age group reported PEG scores 1.6 points higher. There were no differences in pain scores between genders (*p* = 0.20). Participants with comorbidities reported higher pain scores. Participants with three or more comorbidities reported pain scores 0.78 higher compared to those with no comorbidities (*p* < 0.01).

As shown in Table 3, higher SNOT-22 scores were highly, and independently, associated with depression and pain scores (both *p* < 0.01). Adjusting for patient demographics and comorbidities, each point increase in PHQ-9 score was associated with a 1.41 point increase in SNOT-22 score (*p* < 0.01). For a CRS patient with treatable depression, and PHQ-9 score of 10, there was an expected increase of 14 points in the participants’ SNOT-22 score.

**Table 3:** Multivariate analysis of patients’ SNOT-22 scores, adjusting for demographics and comorbidities

Parameter	Estimate	Standard error	F-Statistic *P*-Value
Intercept	16.31	4.04	<0.01
Gender			
Male	Reference		
Female	2.68	2.12	0.20
Age group			
< = 30	6.53	5.31	0.22
31–50	11.04	3.92	<0.01
51–70	7.41	3.58	0.03
70+	Reference		
Number of comorbidities			
0	Reference		
1	1.92	2.87	0.50
2	2.44	3.31	0.46
3	−2.66	4.07	0.51
3+	10.23	3.83	<0.01
Patient-reported outcomes			
PHQ-9	1.41	0.26	<0.01
PEG	2.42	0.50	<0.01

Pain was also highly significantly associated with SNOT-22 score after adjusting for patient demographics and comorbidities (*p* < 0.01). Each point increase in the PEG was associated with a 2.42 point increase in SNOT-22 score. This relationship meant that for a patient with high pain – a PEG score of 3 – there was an expected 7 point increase in the participants’ SNOT-22 score.

The results of Table 4 show the association between participants’ quintile of SNOT-22 sleep score and depression and pain. There was a positive association between sleep scores and depression scores – participants reporting the most severe problems with sleep were several-fold more likely to report moderate depression and high pain as participants with high quality of sleep. Among participants, patients with significant sleep problems were at least 80% likely to have at least moderate depression and high pain.

**Table 4:** PHQ-9 and PEG scores shown by quintile of the sleep function score of SNOT-22

		Depression PHQ-9		Pain PEG	
SNOT-22 sleep function score	N	Mean (SD)	Score > =10 (%)	Mean (SD)	Score > =3 (%)
*Overall*	*261*	*5.2 (5.4)*	*19.2%*	*2.9 (2.7)*	*44.6%*
0–8	62	0.8 (1.6)	1.6%	0.7 (1.1)	6.6%
9–16	61	3.5 (3.9)	8.2%	2.3 (2.4)	37.7%
17–24	75	5.6 (3.6)	12.0%	3.2 (2.0)	53.3%
25–32	46	9.4 (5.7)	45.7%	5.2 (2.7)	76.1%
33–40	17	14.4 (6.1)	82.4%	6.0 (3.2)	82.4%

PHQ-9 scores of ten above and PEG scores of three and above are associated with moderate depression and high pain, respectively

## Discussion

This study focused on patients’ self-reported CRS-related symptoms at the time they were enrolled on the VCH’s wait list for ESS. Our primary interest was in the prevalence of pain and depression, and the relationship between these morbidities and CRS symptoms (as measured by the SNOT-22). Younger participants were more likely to have worse SNOT-22 scores, higher rates of depression and pain after adjusting for other comorbidities. This finding is important, as the relationship between the SNOT-22 score (a condition-specific instrument) and other specific instruments designed to be sensitive to depression and pain, was previously unclear. These are important domains which have been previously reported to be inadequately measured by the SNOT-22 [21], though the effects of which have been suggested elsewhere [11, 13].

The results also found that the worst pain and depression was reported by a subset of patients with severe sleep problems. Indeed, relationships between depression, pain and sleep have been raised by others [15]. The associations between these factors in this study suggest that surgeons could prioritize patients, and improve health status, on the basis of sleep problems alone.

The implications of these findings are substantial. Since this study’s participants’ SNOT-22 scores were comparable to those reported in literature [17, 22, 23], there is very likely to be substantial depression and pain among the population of patients waiting for ESS. In light of these findings, an effective strategy for improving global health could consider complementary non-surgical interventions for depression and pain, focused on patients whose pre-surgical SNOT-22 scores were in the highest quintile, reporting a number of comorbid conditions or reporting poor sleep quality.

There are some limitations of these findings. Study participants were a subset of patients recruited from the VCH wait list, though there was no evidence to believe that patients participating in this study were substantially different from those that declined to participate. Nor do these findings mean that the results are true in all regions of Canada. Finally, as this was a cross-sectional analysis, one cannot draw conclusions regarding possible causal pathways between waiting for ESS for CRS and physical and mental health.

The knowledge generated from this study is timely from a policy perspective, as BC has been directing additional funding into elective surgeries to reduce wait times [24]. The process used to generate patient-reported outcomes data for this study will also have an impact on BC’s Ministry of Health, the funder seeking to improve the patient-centredness of surgical treatment in the province [25], by identifying gaps in medical treatment related to its management of surgical wait lists.

## Conclusion

CRS is a highly prevalent condition and its treatment by ESS is very common. This study underscores that approximately one-fifth of patients waiting for ESS report clinically significant depression and/or pain. While an Otolaryngologist should be attentive to pain and depressive symptoms in all patients, when faced with lengthy waits for ESS, multi-disciplinary treatment, such as involving psychologists or pain management, is indicated for patients who pre-operatively screen with high SNOT-22 scores.

## Additional file

Additional file 1: Appendix A. Surgical codes for defining study cohort. (DOCX 15 kb)

## Abbreviations

BC: British Columbia
BREB: Behavioral Research Ethics Board
CRS: Chronic rhinosinusitis
ESS: Endoscopic sinus surgery
PEG: Pain intensity (P), interference with enjoyment of life (E) and general (G) activity
PHQ-9: Patient Health Questionnaire
PROs: Patient-reported outcomes
SNOT-22: Sino-Nasal Outcomes Test-22
VCH: Vancouver Coastal Health Authority
