# Supplementary material for: Incidence and burden of comorbid pain and depression in patients with chronic rhinosinusitis awaiting endoscopic sinus surgery in Canada
Source: J Otolaryngol Head Neck Surg. 2017 Mar 27;46:23. doi: 10.1186/s40463-017-0205-3 (PMC5369212; doi:10.1186/s40463-017-0205-3)
Supplement: Additional file 1: — Appendix A. Surgical codes for defining study cohort. (DOCX 15 kb) [file 40463_2017_205_MOESM1_ESM.docx]

**Appendix A**. Surgical codes for defining study cohort.

Patients diagnosed with: Chronic sinusitis/polyposis.

Abbreviated surgical codes included one of:

| NASAL CESS ETHMOIDECTOMY W ENDOSCOPY/ANTRAL LAVAGE/NSR |
| --- |
| NASAL CESS ETHMOIDECTOMY W ENDOSCOPY/OSTEOPLASTIC FLAP |
| NOSE ENDOSCOPIC ANTROSTOMY |
| NASAL ETHMOIDECTOMY ENDOSCOPIC COMP W/SPHENOIDECTOMY &  FRONTAL SINUSOTOMY BILATERAL |
| NASAL SINUS MAXILLARY SINOSCOPY W/INTRANASAL SINUS POLYPECTOMY |
| NASAL ETHMOIDECTOMY ENDOSCOPIC COMPLETE BILATERAL |
| NASAL ETHMOIDECTOMY ENDOSCOPIC PARTIAL BILATERAL |

**Appendix B.** Summary statistics of patients waiting for endoscopic sinus surgery, participants and non-participants.

|  |  | Participants | | |  | Non-Participants | |
| --- | --- | --- | --- | --- | --- | --- | --- |
| Characteristic |  | Number | Percent | Mean (SD) SNOT-22 Score |  | Number | Percent |
| *Overall* |  | *261* | *100%* | *42.1 (22.3)* |  | *444* | *100%* |
| Age |  |  |  |  |  |  |  |
| <31 |  | 20 | 43.9% | 43.9 (22.6) |  | 25 | 5.7% |
| 31-50 |  | 74 | 45.7% | 45.7 (21.7) |  | 170 | 38.5% |
| 51-70 |  | 140 | 42.1% | 42.1 (22.9) |  | 208 | 47.0% |
| >70 |  | 27 | 31.1% | 31.1 (18.1) |  | 39 | 8.8% |
| Gender |  |  |  |  |  |  |  |
| Female |  | 130 | 49.8% | 45.2 (21.4) |  | 208 | 46.8% |
| Male |  | 131 | 50.2% | 39.1 (22.8) |  | 236 | 53.2% |
